# A rare case of synchronous anal metastasis from sigmoid adenocarcinoma: case report and literature review

**DOI:** 10.1016/j.ijscr.2025.111505

**Published:** 2025-06-13

**Authors:** Souad Ghattas, Jad El Bitar, Hani Maalouf, Antoine El Asmar, Ziad El Rassi, Wissam Dib

**Affiliations:** a Department of General Surgery – Balamand University -Saint Georges Hospital University Medical Center, Beirut, Lebanon; b Departement of General Surgery - Saint Georges Hospital University Medical Center, Beirut, Lebanon

**Keywords:** Sigmoid adenocarcinoma, Anal metastasis, Epithelial damage, Abdominoperineal resection

## Abstract

Introduction: Anal metastasis from colorectal cancer is extremely rare, since most cases in the literature are associated with a history of anal disease, such as anal fistula, fissure, hemorrhoidectomy, and anastomotic injury.

**Case:**

Herein, we report the case of a 63-year-old male patient, who presented with synchronous anal metastasis from a sigmoid cancer in the absence of epithelial damage treated with trimodality therapy that consisted of neoadjuvant chemoradiotherapy followed by abdominoperineal resection.

**Discussion:**

True cutaneous metastases to the anal skin from colonic carcinomas are exceedingly uncommon and are likely underpinned by diverse mechanisms such as lymphatic dissemination, transperitoneal extension, direct extension, retrograde vascular dissemination, or systemic hematogenous spread. This pattern of metastasis is particularly observed in advanced tumor presentations.

**Conclusion:**

This report guides clinicians to think about this rare type of metastasis. However, more clinical data is necessary to establish treatment and postoperative management plan for anal metastasis derived from colorectal cancer.

## Highlights

• Anal metastasis from colon cancer is a rare manifestation, constituting an atypical trajectory for disease progression.
• Most reported instances of cancer spread describe implantation on compromised anal epithelium, often associated with anal fistulas or fissures.
• This report guides clinicians to think about this rare type of metastasis.
• However, more clinical data is necessary to establish treatment and postoperative management plan for anal metastasis derived from colorectal cancer.

## 1 Introduction

Colorectal cancer is a prevalent malignancy, constituting 10 % of all newly diagnosed cancers annually [1]. In contrast, anal cancer is relatively uncommon, comprising less than 1 % of new cancer diagnoses and accounting for less than 3 % of all gastrointestinal tract tumors [2]. It is noteworthy that within the spectrum of anal cancer cases, anal adenocarcinoma constitutes less than 10 % [2].

The occurrence of synchronous anal metastasis stemming from colon cancer is exceptionally rare. Current literature indicates that most documented cases are associated with a history of anal diseases, such as anal fistula, fissure, hemorrhoidectomy, and anastomotic injury [3]. Remarkably, instances of primary synchronous metastasis without antecedent epithelial damage have been reported in only a solitary case [4].

In this report, we present a unique case involving a patient diagnosed with synchronous adenocarcinoma of the sigmoid, accompanied by anal metastasis. The patient underwent a comprehensive treatment approach, including chemoradiotherapy followed by abdominoperineal resection. This case contributes to the limited body of evidence surrounding such rare occurrences and underscores the complexity of managing synchronous metastases in the absence of preceding epithelial damage.

This case report has been reported in line with the SCARE checklist [5].

## 2 Case

A 63-year-old male patient presented at our hospital with a one-month history of pseudo-obstructive symptoms, characterized by alternating diarrhea and constipation accompanied by abdominal distension. The patient, was a former smoker, nondrinker with a history of hypertension under medical treatment and had no familial background of colon malignancies. Additionally, the patient reported a one-year history of hematochezia and had previously been informed that it was due to hemorrhoids, initiating medical management accordingly.

A prior contrast-enhanced computed tomography of the abdomen and pelvis (CT scan), conducted one month before the presentation, revealed an 8 × 4 cm circumferential enhancing sigmoid lesion located 15 cm from the anal region and clinical examination revealed a perianal friable and ulcerative lesion at 3 o'clock.

**Fig. 1:**
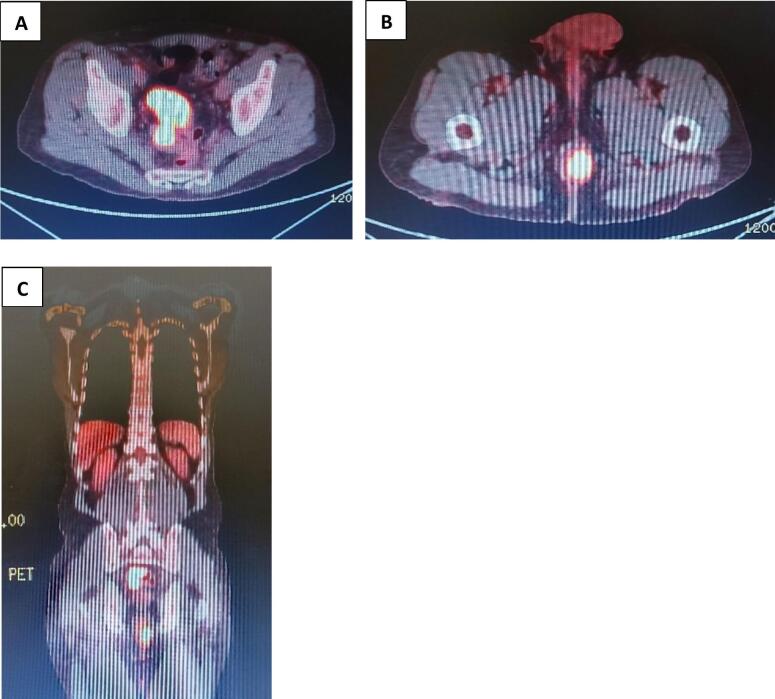
A. PET/CT scan, transversal view showing FDG avid soft tissue masse involving the sigmoid colon B. PET/CT scan, transversal view showing FDG avid soft tissue masse involving the anus C. PET/CT scan, coronal view showing FDG avid soft tissue masses involving the sigmoid colon as well as the anus.

Upon consultation, a colonoscopy was recommended, revealing a pseudo-obstructive circumferential sigmoid mass at 15 cm from the anal verge. The colonoscope could not be advanced proximal to the tumor. Histological analysis confirmed it to be an adenocarcinoma. Two additional sessile polyps in the mid rectum were biopsied and returned benign. A suspicious anal lesion, initially not biopsied, was later biopsied under local anesthesia and diagnosed as a metastatic anal adenocarcinoma of colonic type. Positron emission tomography (PET/CT) showed FDG avid circumferential thickening of the sigmoid colon wall with adjacent fat stranding, extending over more than 7 cm in length with SUV max 31.7 and another FDG soft tissue mass in the anal canal and anus with a larger component on the left, protruding on the anal verge on the left side, extending over approximately 7.5 cm in length with SUV max 22.5. No evidence of FDG avid disease in the rest of the body (Fig. 1).

The patient was diagnosed with synchronous anal metastasis from sigmoid adenocarcinoma. A multidisciplinary team meeting recommended neoadjuvant chemo-radiotherapy after relief of the obstruction. The patient underwent a laparoscopic left lateral colostomy, received 6 cycles of Xeloda (capecitabine) chemotherapy followed by 24 sessions of radiotherapy. Subsequent imaging demonstrated regression of the sigmoid tumor and a reduction in the size of the anal tumor. A follow-up completion colonoscopy revealed a regression of the sigmoid tumor and two benign-looking cecal polyps.

**Fig. 2:**
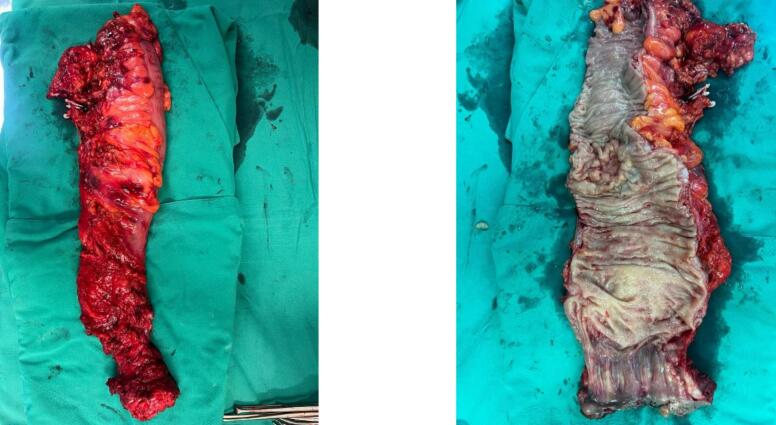
Specimen removed.

Four weeks after completing treatment, the patient underwent a laparoscopic abdominoperineal resection (Fig. 2). The postoperative course was smooth and uneventful. However, one week postoperative, the patient presented with small bowel obstruction. Conservative management was unsuccessful, leading to a diagnostic laparoscopy with adhesiolysis and pelvic biological mesh insertion.

Final histopathology result of the resected specimen confirmed the presence of a Low Grade, Intestinal Type adenocarcinoma of the Sigmoid Colon, invading the muscularis propria and subserosal adipose tissue without lymph node involvement of 33 lymph nodes dissected (ypT3N0) with DNA Mismatch Repair Proteins MLH1,PMS2,MSH2 and MSH6 histology. Sections obtained from the squamous zone of the anal canal also showed viable Well differentiated adenocarcinoma. Tumor was seen invading the smooth muscle layer of the Anal canal with No involvement of the perianal skeletal muscle fibers. The architecture, cytology and immunoprofile were identical to those of the sigmoid adenocarcinoma. These findings are in favor of sigmoid colon adenocarcinoma with extension/mestastasis to the anal cancel rather than primary adenocarcinoa arising from the anal canal glands. Excision was complete.

The patient took adjuvant Xeloda (capecitabine) and is tumor free at 6 months post-operatively.

## 3 Discussion

Colorectal cancer metastasis, while observed in approximately 20 % of patients, typically targets the liver as the predominant site. Conversely, anal metastasis is a rare manifestation, constituting an atypical trajectory for disease progression [1]. The concurrent occurrence of carcinoma in the perianal region and the colon is an exceedingly infrequent phenomenon, with sparse documentation in the scientific literature [6]. Most reported instances of cancer spread describe implantation on compromised anal epithelium, often associated with anal fistulas or fissures [7]. Synchronous adenocarcinomas in the colon and anus without epithelial damage are exceptionally rare occurrences [5].

The underlying mechanistic drivers of this unique clinical presentation remain elusive. True cutaneous metastases to the anal skin from colonic carcinomas are exceedingly uncommon and are likely underpinned by diverse mechanisms such as lymphatic dissemination, transperitoneal extension, direct extension, retrograde vascular dissemination, or systemic hematogenous spread. This pattern of metastasis is particularly observed in advanced tumor presentations [8].

Immunohistochemical staining utilizing CK7 and CK20 biomarkers is a standard approach to corroborate the metastatic origin of anal adenocarcinoma. Colorectal adenocarcinomas typically exhibit robust immunoreactivity for CK20, while anal glands manifest immunoreactivity with CK7 antibodies. Notably, intestinal metastases often present as submucosal tumors, emanating from submucosal vessels and predominantly situated within the submucosa and muscularis propria [9].

Preoperative colonoscopy assumes a pivotal role as a standard diagnostic practice, especially for identifying synchronous colonic adenocarcinoma, as it significantly influences the surgical plan [11]. Conversely, the identification of synchronous anal adenocarcinoma alongside colonic adenocarcinoma is a rare occurrence, particularly in the absence of epithelial damage. A solitary documented case of rectal adenocarcinoma with synchronous anal metastasis has been recorded, further highlighting the scarcity of such presentations [5]. Other reported cases involve metachronous anal metastasis from sigmoid colon cancer, with most instances featuring tumor implantation on compromised epithelium such as anal fistula, anal fissure, or post-surgical anal trauma [9].

Despite considerable advancements in the management of primary and metastatic colorectal cancer, the rarity of anal metastasis has precluded the establishment of a standardized treatment protocol. Existing literature presents a dichotomy between radical abdominoperineal resection (APR) and sphincter-sparing surgery [13]. Tailoring treatment approaches to individual patients is imperative, considering factors such as comorbidities and tumor characteristics including location, size, dissemination, and technical feasibility [13]. In the case under consideration, after exhaustive deliberations in a multidisciplinary setting, a preoperative regimen of chemoradiation followed by abdominoperineal resection was deemed the most appropriate course of action. This decision was informed by evidence of sphincter involvement and the impracticability of local excision. Comprehensive analysis from the National Cancer Data Base, encompassing a substantial cohort of patients with anal adenocarcinoma, supports the application of trimodality therapy involving neoadjuvant chemoradiotherapy followed by abdominoperineal resection, correlating with enhanced overall survival outcomes [14].

## 4 Conclusion

Our encounter with a highly unusual case involving synchronous sigmoid adenocarcinoma and anal metastasis in the absence of epithelial damage serves as a compelling illustration of the rarity of this metastatic pattern. This report serves as an instructive reference for clinicians, prompting consideration of such atypical metastatic scenarios in colorectal cancer patients.

The scarcity of reported cases underscores the challenge in formulating a standardized management approach, as the limited evidence base hampers the establishment of clear therapeutic guidelines. The dearth of clinical data necessitates a comprehensive accumulation of cases to inform treatment strategies and postoperative management plans specific to colorectal cancer-derived anal metastasis. This case contributes to the growing awareness of this rare phenomenon and highlights the imperative for further clinical investigation to delineate optimal therapeutic interventions and refine the overall management of such intricate cases.

## CRediT authorship contribution statement

Souad Ghattas (First Author, conception of the work, design of the work, revising the work critically for important intellectual content), Jad El Bitar (co-Author, review of literature, draft manuscript, revising the work critically for important electual content), Hani Maalouf (co-Author, review of literature, draft manuscript, revising the work critically for important electual content), Antoine El Asmar (co-Author, supervision, validation, project administration, conception of the work,revising the work critically for important intellectual content), Ziad El Rassi (co-Author, supervision, validation, project administration,conception of the work,revising the work critically for important intellectual content, final approval of the version to be published project administration and corresponding author.), Wissam Dib (Corresponding Author, supervision, validation, project administration,conception of the work,revising the work critically for important intellectual content, final approval of the version to be published project administration and corresponding author.)

## Informed consent

Written informed consent was obtained from the patient for publication and any accompanying images. A copy of the written consent is available for review by the Editor-in-Chief of this journal on request.

## Ethical approval

Case report approved for publishing by the ethical committee at Saint Georges Hospital University Medical Center, and Head of General Surgery division, Beirut, Lebanon, 2024.

## Guarantor

Souad Ghattas

## Provenance and peer review

Not commissioned, externally peer-reviewed.

## Source of funding

None.

## Declaration of competing interest

The authors report no conflicts of interest.

## Data Availability

All Data are available upon request at the Department of General Surgery, Saint Georges Hospital University Medical Center, Beirut, Lebanon where the work was done.
